# Coffee extraction kinetics in a well mixed system

**DOI:** 10.1186/s13362-016-0024-6

**Published:** 2016-06-30

**Authors:** Kevin M Moroney, William T Lee, Stephen BG O’Brien, Freek Suijver, Johan Marra

**Affiliations:** 1 MACSI, Department of Mathematics and Statistics, University of Limerick, Limerick, Ireland; 2 Philips Research, Eindhoven, The Netherlands

**Keywords:** double porosity model, coffee extraction kinetics, leaching, solid-liquid extraction, matched asymptotic expansions

## Abstract

The extraction of coffee solubles from roasted and ground coffee is a complex operation, the understanding of which is key to the brewing of high quality coffee. This complexity stems from the fact that brewing of coffee is achieved through a wide variety of techniques each of which depends on a large number of process variables. In this paper, we consider a recent, experimentally validated model of coffee extraction, which describes extraction from a coffee bed using a double porosity model. The model incorporates dissolution and transport of coffee in the coffee bed. The model was shown to accurately describe extraction of coffee solubles from grains in two situations: extraction from a dilute suspension of coffee grains and extraction from a packed coffee bed. The full model equations can only be solved numerically. In this work we consider asymptotic solutions, based on the dominant mechanisms, in the case of coffee extraction from a dilute suspension of coffee grains. Extraction in this well mixed system, can be described by a set of ordinary differential equations. This allows analysis of the extraction kinetics from the coffee grains independent of transport processes associated with flow through packed coffee beds. Coffee extraction for an individual grain is controlled by two processes: a rapid dissolution of coffee from the grain surfaces in conjunction with a much slower diffusion of coffee through the tortuous intragranular pore network to the grain surfaces. Utilising a small parameter resulting from the ratio of these two timescales, we construct asymptotic solutions using the method of matched asymptotic expansions. The asymptotic solutions are compared with numerical solutions and data from coffee extraction experiments. The asymptotic solutions depend on a small number of dimensionless parameters, so the solutions facilitate quick investigation of the influence of various process parameters on the coffee extraction curves.

## Introduction

Coffee is a popular beverage prepared from the beans (seeds) of the coffee plant. The consumption of coffee, which has been increasing in recent years, is on a global scale. In 2010 coffee production reached 8.1 million tonnes worldwide, which represents more than 500 billion cups [1]. Typically coffee preparation involves three main stages. First the raw coffee beans are roasted. Following this the roasted beans are ground or milled to facilitate a faster extraction during the final brewing stage. The brewing stage involves the leaching of coffee solubles from the roasted and ground coffee grains with hot water. Generally the extract is filtered to remove undissolved solids from the final coffee beverage before consumption. There are a wide variety of techniques used in the brewing stage. These brewing methods fall into three broad categories: decoction methods, infusion methods and pressure methods. A large number of these techniques is described in [2, 3]. Despite the wide variety of methods available to brew coffee, each method relies on solid-liquid extraction of coffee solubles from coffee grains with hot water as the central operation. Even in its simplest manifestation the brewing of coffee is a complex operation which is dependent a large number of process variables. Important parameters include the brew ratio (dry coffee mass to water volume used), grind size and distribution, brewing time, water temperature, agitation, water quality and uniformity of extraction [2, 4].

A fundamental goal for the manufacturers of coffee brewing machines and end users of their products is to be able to consistently brew the highest quality coffee possible. This presents a number of challenges. First of all coffee quality is difficult to define and to some degree a matter of taste. In addition to this, even if the composition of the extracted coffee solution can be identified for different tastes, the coffee machine should be able to change the brewing parameters to move between these ideal compositions. To achieve this goal, both a model relating the final composition of the brewed coffee to the process parameters, and a measure of the quality of the brewed coffee, are required. The complex chemistry of coffee makes it difficult to find correlations between the individual chemical constituents of the extracted coffee solubles and the quality of the final beverage. In the absence of such a description, a set of standards, summarised in the coffee brewing control chart [4], is often used as a simple measure of coffee quality. This chart gives target ranges for the brew strength and extraction yield of the coffee based on preferences recorded in organised taste tests. Brew strength is defined as the ratio of the mass of dissolved coffee in the beverage to volume. Extraction yield is the percentage of dry coffee grind mass that has been extracted as solubles into the water. Brew strength and extraction yield are related by the brew ratio. Given that the most widely used measure of coffee quality considers coffee as a single component, it seems logical to model extraction based on a single coffee constituent [5]. Clearly modelling of the coffee concentration (brew strength) of the final beverage for a particular brewing apparatus, with the process parameters as inputs, could be a valuable tool in order to choose the optimal set of parameters to achieve a targeted coffee quality on the coffee brewing control chart. To achieve this goal for any brewing apparatus, an accurate physical description of the extraction kinetics of coffee solubles from the coffee grains is a key requirement.

The physics of coffee extraction from various coffee brewing systems has been the subject of some investigation and modelling over the years. Industrial scale coffee extraction to produce instant coffee has been studied with the aim of optimising the design of these systems. Early work [6, 7] looked at models of coffee extraction in large packed columns, called diffusion batteries, with the aim of extracting highly concentrated solution. In these systems, extraction was performed by forcing hot water through these columns to extract solubles. In order to achieve the maximum possible concentration in the extract, extraction was performed at high temperatures and columns were connected consecutively in a series. Much of this work is summarised in [8]. At a smaller scale, domestic and catering brewing of coffee has also been subject of some investigation. Experiments on the operation and efficiency of the stove-top of Moka pot are detailed in [9, 10]. Fasano *et al.* have developed some very general multiscale models of coffee extraction in different situations, with a particular focus on the espresso coffee machine [11–16]. The influence of some brewing parameters on coffee extraction was investigated by Voilley and Simatos [17]. A number of different extraction experiments was conducted on a well-mixed system of coffee grounds and water. The response of brew strength to variations in process parameters such as brewing time, granule size, brew ratio and water temperature was considered. A simple model was used to describe the variations of brew strength during the experiments. The model assumed the coffee grains were spherical and suspended in a homogeneous system. Extraction was then modelled as diffusion of a single component from a sphere with a diameter equal to the mean grain diameter. The model was fitted to the data using the diffusion coefficient as a fitting parameter and was found to provide reasonable agreement with the experimental extraction curve. It was noted, however, that the initial extraction proceeded at a faster rate in the experiment compared to the model extraction curve.

**Figure 1:**
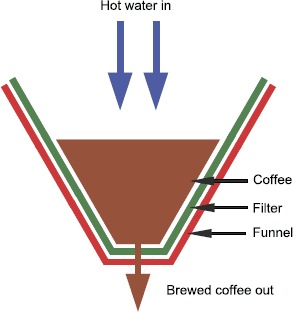
**Drip filter brewing system.** Drip filter brewing involves pouring hot water over coffee grounds in a filter. As the water flows through the bed soluble coffee components are leached from the grains. Any undissolved solids in the fluid are filtered from the extract as the liquid leaves the filter.

More recently, the drip filter brewing system has been the subject of increased attention. In this brewing technique hot water is poured over coffee grounds contained in a filter which is often conical in shape. The hot water flows down through the coffee bed under the influence of gravity, leaching soluble content. The only impediment to flow is the resistance provided by the porous bed and the filter. A schematic of this process is shown in Figure 1. The drip filter brewing system was analysed by a group of applied mathematicians at the ESGI 87 study group with industry at the University of Limerick [18]. The problem was brought to the study group by coffee researchers at Philips Research in Eindhoven. Some of the aspects considered included the evolution of the shape of the coffee bed during brewing, correlations observed between the final bed shape and the quality of the brewed coffee and the use of a single jet or multiple jets (shower head) to deliver water to the coffee bed. Following this, a paper by one of the study group participants [19], analysed gravity driven flow in a conical filter, similar to that used in a drip filter machine, and constructed a mathematical model describing the flow. In a recent paper, Moroney *et al.* [5] presented a new multiscale model of coffee extraction from a coffee bed. The model is derived from first principles, by forming balance equations for coffee solubles and water in the different phases in the bed, at different length scales. The parameters in the macroscale model are related to the microscale parameters in the coffee bed by a volume averaging procedure. Transport of water or coffee solubles across the phase boundaries on the microscale appear as source terms on the macroscale. The general model is then specialised based on observations in coffee extraction experiments. In particular it was noted that extraction seemed to proceed in two stages; an initial rapid extraction over a short period, followed by a significantly slower extraction during the rest of the brewing period. This phenomenon was explained by assuming that the fast initial extraction was due to a reduced mass transfer resistance in fine particles and the broken cells on the surfaces of grains following grinding. The slower extraction was assumed to occur due to a much larger mass transfer resistance in the intact coffee cells in the kernels of larger coffee grains. The model was specialised to describe coffee extraction in two experimental situations: extraction from a dilute suspension of coffee grains in water and extraction from a packed coffee bed. Numerical simulations of the model equations showed that the model could quantitatively reproduce the extraction profiles of these experiments for one fine coffee grind and one coarse coffee grind considered.

In this paper the coffee extraction model from [5] is analysed in the case of the extraction from a dilute suspension of coffee grains. The analysis in the case of extraction from a packed coffee bed is the subject of a separate paper [20]. Analysing extraction from a well-mixed, dilute suspension of coffee grains is of interest, as it allows us to investigate the extraction kinetics from the coffee grains, independent of the complications introduced by the advection and mechanical dispersion occurring in pressurised flow through a tortuous packed coffee bed. The general model from [5] will be introduced and specialised to describe extraction from a dilute suspension of coffee grains. The dominant mechanisms during the extraction are then identified by non-dimensionalising the equations. Approximate solutions are formed based on the dominant processes during different stages of the extraction. The approximate solutions are compared with numerical solutions of the system and also with experimental data presented in [5].

## General coffee extraction model

**Figure 2:**
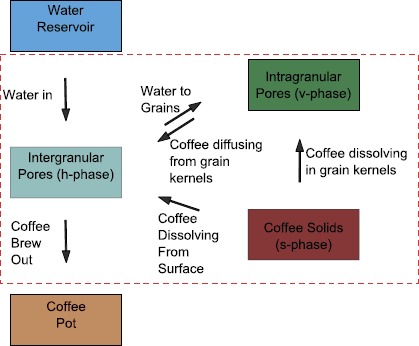
**Coffee and fluid transfers in the coffee bed.** The diagram shows the transfers of water and coffee which are described by the coffee extraction model presented in [5]. Boundary conditions must be prescribed to describe any transfers of fluid or solubles in or out of the bed where necessary.

**Figure 3:**
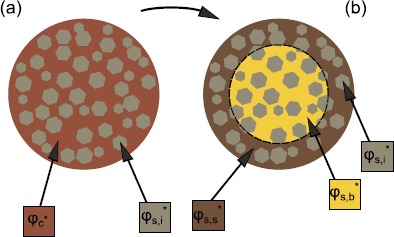
**Distribution of coffee in a coffee grain.** The coffee grains make up a volume fraction $1-\phi_{h}$ of the bed. The grain volume consists of (intragranular) pores of volume fraction $\phi _{v}^{*}$ and solids of volume fraction $\phi_{s}^{*}=1-\phi_{v}^{*}$. The schematic shows how the solid volume fraction is divided (intragranular pores are not represented for clarity). In diagram **(a)**, the solid is divided into a soluble volume fraction $\phi_{c}^{*}$ and an insoluble volume fraction $\phi_{s,i}$. In diagram **(b)**, this soluble volume fraction is broken into a volume fraction near the surface of grains $\phi_{s,s}^{*}$ and a volume fraction in the kernels (bulk) of grains $\phi_{s,b}^{*}$.

The general coffee extraction model outlined here is described in detail in [5]. The full model will not be analysed in this paper, but it is useful to summarise the model before specialising to the case of interest here. The coffee bed is represented using a double porosity model. This means that the coffee bed consists of grains which are themselves porous. Temperature variations during brewing are considered to be small, so isothermal conditions are assumed, and the variation in any temperature dependent parameters is considered negligible. At the macroscale the model consists of conservation equations for coffee and liquid in three phases. The phase consisting of the pores between the coffee grains (intergranular pores) is called the *h*-phase. The pores inside the grains (intragranular pores) make up the *v*-phase. Finally the solid coffee cellular matrix in the grains is called the *s*-phase. The main transfers of coffee solubles and fluid between phases in the coffee bed are illustrated in Figure 2. The coffee concentrations (mass per unit volume) in each of the phases are denoted by $c_{h}^{*}$, $c_{v}^{*}$ and $c_{s}$. The solid coffee matrix density, $c_{s}$, is assumed constant. Variables in the dimensional model which will appear in scaled form in the dimensionless model are denoted by an asterisk. Note that this includes some intrinsically dimensionless variables which are normalised in the dimensionless model. The porosity or volume fraction of the intergranular pores is denoted by $\phi_{h}$ and is assumed constant. The volume fraction of the grains $(1-\phi_{h})$ is divided into two domains. The intragranular pores have a volume fraction (of the total grain volume) of $\phi_{v}^{*}$, while the solid coffee matrix has a grain volume fraction of $\phi_{s}^{*} = 1-\phi_{v}^{*}$. The actual solid coffee volume fraction is further divided into three parts as illustrated in Figure 3. The volume fraction of coffee which is insoluble under the conditions in the coffee bed is denoted by $\phi_{s,i}$ and may depend on water temperature, coffee grind distribution and other variables. Experiments in [5] show that for water at 90°C the extractable mass in coffee grains can vary from 28% for coarse grinds to 32% for fine grinds of the same coffee. The soluble coffee grain volume fraction is split into two parts according to its position in the coffee grains. Fines, which are broken cell fragments produced during grinding of the coffee beans, can account for a significant volume of the coffee grind distribution, particularly for finer grinds. Following grinding the surfaces of the coffee grains consist mainly of broken coffee cells. Coffee in each of these regions in the coffee bed is expected to have a significantly lower mass transfer resistance relative to that in intact cells in the kernels of larger grains. The volume fraction of this coffee is denoted $\phi_{s,s}^{*}$. The intact cells in the kernels of larger grains have a much higher mass transfer resistance. The volume fraction of this coffee is denoted $\phi _{s,b}^{*}$. Thus the volume fraction of soluble coffee in the coffee grains is $\phi_{c}^{*} = \phi_{s,s}^{*} + \phi_{s,b}^{*}$. It is also useful to track the fraction of the original amount of coffee (in the dry coffee grains) present in the grains surfaces and in the grain kernels at a given time during brewing. The volume fractions of coffee in the dry coffee grains, grain surfaces and grain kernels are given by $\phi_{cd}$, $\phi_{s,sd}$ and $\phi_{s,bd}$ respectively. The fractions of the original amount of coffee left on the grain surfaces and in the grain kernels are denoted by $\psi_{s}^{*}$ and $\psi_{v}^{*}$.

In extractions involving flow through a bed of grains the surface area of the coffee grain distribution is a key parameter influencing the flow. The surface area is also key to extraction of coffee during the brewing process. The specific surface area of the entire grain distribution, which can influence flow and extraction of coffee from grain surfaces, is described using the Sauter mean diameter $k_{sv1}$. This is the diameter of a spherical particle which has the same surface area to volume ratio as the entire grind size distribution [21]. The specific surface area of larger grains (diameter larger than 50 μm), which influences extraction from the kernels of these larger grains, is described using the Sauter mean diameter $k_{sv2}$. The effective diffusion coefficient of coffee in water is given by $D_{h}$ in the *h*-phase and $D_{v}$ in the *v*-phase. The effective diffusion distance between the *v*-phase and the *h*-phase is given by $l_{l}$. The average coffee cell radius in the grains is given by *m*. It is assumed that there is some coffee concentration $c_{\mathrm {sat}}$, which is the concentration in the liquid phase that would be in equilibrium with the concentration in the solid. Here we take it to be the maximum solubility of coffee in the liquid. Mechanical dispersion can be an important factor in flows in porous media [22]. The dispersion tensor is denoted by $\tilde{D}^{b}$. Other important parameters necessary to complete the description include the fluid density *ρ*, the dynamic viscosity of the fluid *μ* and the shape factor *κ* from the Kozeny-Carman equations. The system of equations in [5] included a term for transfer of fluid (and coffee solubles as a result) between the *h*-phase and *v*-phase due to differences in pressures. This correction is difficult to model accurately without further experimental insight and is thought to occur much faster than other bed processes. For this reason these terms are neglected in the presentation of the equations here. Neglecting these terms, the general mathematical model becomes

1 $$\begin{aligned}& \phi_{h}\frac{\partial c_{h}^{*}}{\partial t^{*}} = \frac {k_{sv1}^{2}\phi_{h}^{3}}{36 \kappa\mu(1-\phi_{h})^{2}}\boldsymbol{ \nabla}^{*}\cdot\bigl(c_{h}^{*} \bigl(\boldsymbol{ \nabla}^{*} p_{h}^{*} + \rho g\bigr)\bigr)+ \phi_{h}^{\frac{4}{3}} D_{h} {\boldsymbol{ \nabla}^{*}}^{2}c_{h}^{*} \\& \hphantom{\phi_{h}\frac{\partial c_{h}^{*}}{\partial t^{*}} ={}}{} + \phi_{h} \tilde{D}^{b}\cdot{\boldsymbol{ \nabla}^{*}}^{2}c_{h}^{*}- (1-\phi_{h}){ \phi_{v}^{*}}^{\frac{4}{3}} D_{v} \frac{6}{k_{sv2} l_{l}}\bigl(c_{h}^{*} - c_{v}^{*} \bigr) \\& \hphantom{\phi_{h}\frac{\partial c_{h}^{*}}{\partial t^{*}} ={}}{}+ (1-\phi_{h})\frac{12 D_{h} \phi_{cd}}{k_{sv1} m}\bigl(c_{\mathrm{sat}} - c_{h}^{*}\bigr)\psi_{s}^{*} , \end{aligned}$$

2 $$\begin{aligned}& {\boldsymbol{ \nabla}^{*}}^{2} p_{h}^{*}=0, \end{aligned}$$

3 $$\begin{aligned}& \frac{\partial}{\partial t^{*}}\bigl( \phi_{v}^{*} c_{v}^{*} \bigr) = {\phi _{v}^{*}}^{\frac{4}{3}} D_{v} \frac{6}{k_{sv2} l_{l}}\bigl(c_{h}^{*} - c_{v}^{*} \bigr) + \frac{12 \phi_{cd}D_{v}}{m^{2}}\bigl(c_{\mathrm{sat}} - c_{v}^{*} \bigr)\psi_{v}^{*}, \end{aligned}$$

4 $$\begin{aligned}& \frac{\partial\phi_{v}^{*}}{\partial t^{*}} = - \frac{1}{r_{s}} \frac {\partial\psi_{s}^{*}}{\partial t^{*}} - \frac{1}{r_{v}} \frac {\partial\psi_{v}^{*}}{\partial t^{*}} , \end{aligned}$$

5 $$\begin{aligned}& \frac{\partial\psi_{s}^{*}}{\partial t^{*}} = -\frac{12 D_{h} \phi _{cd}}{k_{sv1} m} \biggl(\frac{c_{\mathrm{sat}} - c_{h}^{*}}{c_{s}} \biggr)r_{s} \psi_{s}^{*}, \end{aligned}$$

6 $$\begin{aligned}& \frac{\partial\psi_{v}^{*}}{\partial t^{*}} = -\frac{12 D_{v} \phi _{cd}}{m^{2}} \biggl(\frac{c_{\mathrm{sat}} - c_{v}^{*}}{c_{s}} \biggr)r_{v} \psi_{v}^{*}. \end{aligned}$$

The constants $r_{s}$ and $r_{v}$ are the reciprocals of $\phi_{s,sd}$ and $\phi_{s,bd}$ respectively. Boundary conditions need to be prescribed depending on the geometry of the coffee bed considered. Initial conditions need to be determined or inferred from experiment once the bed is saturated with water, following the initial addition of water to the dry coffee bed. Alternatively, modelling of the unsaturated flow during the initial infiltration of water could be used.

A complete description of equations (1)-(6) is presented in [5]. The exact form of the coefficients in terms of the process parameters is arrived at through a volume averaging procedure. This process is detailed in the appendices of [5]. For the purposes of this paper, it is sufficient to point out the meaning of each of the terms in the equations, in the context of the transport process taking place in the system. Equation (1) describes how the concentration of coffee in the interstitial fluid (intergranular pores) is changing during brewing. The first term on the right-hand side of the equation describes the advection of coffee in the fluid flow based on Darcy’s Law and the Kozeny-Carman equations. The second and third terms describe diffusion and mechanical dispersion of the coffee solubles relative to the flow. The fourth term describes transport of coffee into the interstitial fluid from the fluid in the intact cells in the grain kernels. The final term describes the rapid dissolution of coffee from the fines and broken cell surfaces directly into the interstitial fluid. Equation (2) gives the equation for flow of the fluid in the intergranular pores in a coffee bed according to Darcy’s Law. Equation (3) describes the changes in the quantity of coffee solubles in the intragranular pores during brewing. Note here that along with changes in the coffee solubles concentration $c_{v}^{*}$, the intragranular porosity $\phi_{v}^{*}$ may also change as coffee dissolves from the cell walls within the grains. On the right-hand side of the equation, the quantity of coffee in the intragranular pores is decreased by slow diffusion of coffee out of the grains (first term), but increased by coffee dissolving from the cell walls (second term). The diffusion length scale has been adjusted for grain tortuosity $\tau_{v}$, using the functional relationship $\tau_{v} = {\phi_{v}^{*}}^{-\frac{1}{3}}$. Equations (5) and (6) keep track of the fraction of the initial amount (in dry coffee) of coffee present on the grain surfaces and in the grain kernels at a given time respectively. The terms on the right-hand side of these equations model the dissolution of solid coffee in these two regions. As this coffee dissolves the intragranular porosity grows from its initial value. This increase in intragranular porosity is described by (4). In the following section this dimensional system of equations will be specialised to model experimental results for extraction of coffee from a dilute suspension of coffee grains. The model will be non-dimensionalised and analysed to develop approximate solutions.

## Coffee extraction model for coffee grains in a fixed volume of water

One of the experiments described in [5] involves mixing 60 g of coffee grind with 0.5 L of hot water and measuring the concentration of the extracted species as a function of time. In this situation the coffee extraction model can be simplified. Firstly it is assumed that the solution in the liquid outside the grains (*h*-phase) is well mixed, since only the average concentration is measured anyway. As a result, the solution in the h-phase has uniform concentration. This means that the spatial derivatives in the model disappear and we have the following system of ordinary differential equations:

7 $$\begin{aligned}& \phi_{h}\frac{d c_{h}^{*}}{d t^{*}} = - \alpha^{*}(1-\phi _{h}){\phi_{v}^{*}}^{\frac{4}{3}} D_{v} \frac{6}{k_{sv2} l_{l}}\bigl(c_{h}^{*} - c_{v}^{*}\bigr) \\& \hphantom{\phi_{h}\frac{d c_{h}^{*}}{d t^{*}} ={}}{} + \beta^{*}(1-\phi_{h})\frac{12 D_{h} \phi_{cd}}{k_{sv1} m} \bigl(c_{\mathrm{sat}} - c_{h}^{*}\bigr)\psi_{s}^{*}, \end{aligned}$$

8 $$\begin{aligned}& \frac{d c_{v}^{*}}{d t^{*}} = \alpha^{*}{\phi_{v}^{*}}^{\frac {1}{3}} D_{v} \frac{6}{k_{sv2} l_{l}}\bigl(c_{h}^{*} - c_{v}^{*}\bigr) +\gamma ^{*}{\phi_{v}^{*}}^{-1} \frac{12 \phi_{cd}D_{v}}{m^{2}}\bigl(c_{\mathrm{sat}} -c_{v}^{*}\bigr) \psi_{v}^{*} \\& \hphantom{\frac{d c_{v}^{*}}{d t^{*}} ={}}{}-\frac{c_{v}^{*}}{\phi_{v}^{*}}\frac{d \phi_{v}^{*}}{d t^{*}}, \end{aligned}$$

9 $$\begin{aligned}& \frac{d \phi_{v}^{*}}{d t^{*}} = - \frac{1}{r_{s}} \frac{d \psi _{s}^{*}}{d t^{*}} - \frac{1}{r_{v}} \frac{d \psi_{v}^{*}}{d t^{*}} , \end{aligned}$$

10 $$\begin{aligned}& \frac{d \psi_{s}^{*}}{d t^{*}} = -\beta^{*}\frac{12 D_{h} \phi _{cd}}{k_{sv1} m} \biggl( \frac{c_{\mathrm{sat}} - c_{h}^{*}}{c_{s}} \biggr)r_{s} \psi_{s}^{*}, \end{aligned}$$

11 $$\begin{aligned}& \frac{d \psi_{v}^{*}}{d t^{*}} = -\gamma^{*}\frac{12 D_{v} \phi _{cd}}{m^{2}} \biggl( \frac{c_{\mathrm{sat}} - c_{v}^{*}}{c_{s}} \biggr)r_{v} \psi_{v}^{*}, \end{aligned}$$

12 $$\begin{aligned}& t^{*}>0, \end{aligned}$$

13 $$\begin{aligned}& c_{h}^{*}(0) = c_{h0}^{*}, \qquad c_{v}^{*}(0) = c_{v0}^{*}, \qquad \phi _{v}^{*}(0) = \phi_{v0}^{*}, \qquad \psi_{s}^{*}(0) = \psi_{s0}^{*},\qquad \psi_{v}^{*}(0) = \psi_{v0}^{*}. \end{aligned}$$

The last term now appearing on the right-hand side of equation (8) is a volume correction term to allow for changes in the intragranular porosity. Additional parameters $\alpha^{*}$, $\beta ^{*}$ and $\gamma^{*}$ are introduced here to fit the model to experiment. These parameters are necessary due to the difficulty in obtaining accurate values for some of the model parameters. For example the diffusion coefficient used for coffee solubles will be for that of caffeine in water. However caffeine diffusivity may differ from the average coffee diffusivity in water and the extraction of many coffee components from the grains cellular matrix is hindered to a greater degree than that of caffeine [23]. Parameters relating to coffee grain properties, such as the cell radius and external and particularly internal specific surface areas are similarly difficult to measure. Thus fitting parameters are required. Clearly to solve this system we need to prescribe initial conditions for each of the dependent variables. This model does not include the addition of the water to the dry coffee grains or the infiltration of water into the intragranular pores. Rather it is assumed that this infiltration occurs quickly and the model starts with the grains saturated with water. This raises the question as to what initial conditions we should use for the model. How much of the coffee has dissolved during the addition of water to the coffee and the infiltration of water into the coffee grains? In the absence of experimental data or separate modelling of this initial filling stage we will need to prescribe appropriate initial conditions. The simplest choice would be to assume that no coffee dissolves during this filling stage so that all initial concentrations are zero but this may not be the most useful.

To reduce the steps we have to take when non-dimensionalising the problem it is useful to make some simplifying steps at this stage based on our knowledge of the problem. First of all the sum of the intragranular pore volume fraction, and the soluble coffee volume fraction is conserved. This is described by equation (9). Integrating this equation with respect to time we obtain

14 $$ \phi_{v}^{*}(t) + \frac{1}{r_{s}}\psi_{s}^{*}(t) + \frac{1}{r_{v}}\psi _{v}^{*}(t) = K, $$

with *K* a constant. We can determine this constant by denoting the final intragranular porosity when all soluble coffee has been dissolved as $\phi_{v}^{\infty}$. The value of $\phi_{v}^{\infty}$ can be estimated from data on the percentage of soluble coffee for a particular coffee grind. Thus we have

15 $$ \phi_{v}^{*}(t) =\phi_{v}^{\infty}- \frac{1}{r_{s}}\psi_{s}^{*}(t) - \frac{1}{r_{v}} \psi_{v}^{*}(t). $$

Using this equation and (9) we can eliminate $\phi _{v}^{*}$ from the model system of equations. We can further simplify the system by noting that the change in intragranular porosity during extraction is small relative to the final (or initial) value of intragranular porosity. More explicitly $\phi_{s,sd}+\phi_{s,bd}= r_{s}^{-1}+r_{v}^{-1} \ll\phi_{v}^{\infty}$. Thus we make the approximation that $\phi_{v}^{*} \approx\phi_{v}^{\infty}$ in the equations. Initial conditions should be adjusted to account for this. Also the volume correction term now drops out of equation (8).

Finally we consider the fitting parameters $\alpha^{*}$, $\beta^{*}$ and $\gamma^{*}$. Experimental data for $c_{h}^{*}$ obtained in [5] reveals a rapid initial extraction followed by a much slower extraction. We can use this data to fit $\alpha^{*}$ and $\beta^{*}$. As dissolution of coffee within the grains and diffusion of coffee from the grains occur consecutively, fitting the parameter $\alpha^{*}$ rather than $\gamma^{*}$ corresponds to the assumption that diffusion of coffee from the grains is the rate controlling step in this series. The fitting parameter $\gamma^{*}$ for the rate of dissolution of coffee from the cell walls within the grains cannot be determined independently from $\alpha^{*}$ from experimental data available in [5]. This is because the intragranular concentration $c_{v}^{*}$ is not measured experimentally. A reasonable guess however may be that it has a value similar to the surface dissolution fitting parameter $\beta^{*}$. In reality, due to a larger surface area per unit volume within the coffee grains, it is likely $\gamma^{*} > \beta^{*}$. The timescale on which surface dissolution occurs is much shorter than that of diffusion of coffee from the grain kernels. Also based on the assumption $\gamma^{*} > \beta^{*}$, dissolution from cell walls within the grains occurs on a similar timescale to surface dissolution. Thus diffusion through grain cellular structure is the rate controlling step in the extraction of solubles from the intact cells in the grain kernels. This suggests that we can approximate these processes by assuming that all the coffee in the grain kernels dissolves very quickly initially into the intragranular pores and extraction is just controlled from there. This of course means that the concentration in the intragranular pores and the intragranular porosity will be incorrect at least for a short period initially but in any case we have no data for comparison. The approximation has the advantage of giving us easy to calculate initial conditions. To ensure conservation of coffee during extraction we need to be careful when prescribing initial conditions. Finally we assume that initially none of the coffee on the grain surfaces has dissolved. Based on this discussion we choose the following dimensional initial conditions:

16 $$\begin{aligned}& c_{h}^{*}(0) = 0, \qquad c_{v}^{*}(0) = \frac{\phi_{s,bd}}{\phi_{v}^{\infty }}c_{s}, \end{aligned}$$

17 $$\begin{aligned}& \psi_{s}^{*}(0) = 1, \qquad \psi_{v}^{*}(0) = 0. \end{aligned}$$

Since $\psi_{v}^{*}(0) = 0$, we have reduced the system to the following three ordinary differential equations:

18 $$\begin{aligned}& \frac{d c_{h}^{*}}{d t^{*}} = - \alpha^{*}\frac{1-\phi_{h}}{\phi _{h}}{ \phi_{v}^{\infty}}^{\frac{4}{3}} D_{v} \frac{6}{k_{sv2} l_{l}}\bigl(c_{h}^{*} - c_{v}^{*} \bigr) \\& \hphantom{\frac{d c_{h}^{*}}{d t^{*}} ={}}{}+ \beta^{*}\frac{1-\phi_{h}}{\phi_{h}} \frac{12 D_{h} \phi _{cd}}{k_{sv1} m} \bigl(c_{\mathrm{sat}} - c_{h}^{*}\bigr)\psi_{s}^{*}, \end{aligned}$$

19 $$\begin{aligned}& \frac{d c_{v}^{*}}{d t^{*}} = \alpha^{*}{\phi_{v}^{\infty}}^{\frac {1}{3}} D_{v} \frac{6}{k_{sv2} l_{l}}\bigl(c_{h}^{*} - c_{v}^{*}\bigr), \end{aligned}$$

20 $$\begin{aligned}& \frac{d \psi_{s}^{*}}{d t^{*}} = -\beta^{*}\frac{12 D_{h} \phi _{cd}}{k_{sv1} m} \biggl( \frac{c_{\mathrm{sat}} - c_{h}^{*}}{c_{s}} \biggr)r_{s} \psi_{s}^{*}. \end{aligned}$$

### Non-dimensionalisation

There are three main timescales evident in the equations: The timescale on which the coffee dissolves within the grains, the timescale on which coffee dissolves from the grain surfaces and finally the timescale on which coffee diffuses from the intragranular pores to the surrounding fluid. We have already made the assumption that the diffusion of coffee from the intragranular pores occurs on a much longer timescale than dissolution of coffee within the grains. On the basis that these processes occur consecutively, only diffusion of coffee from the intragranular pores is considered as the rate limiting step. Thus there are two remaining timescales to consider. We denote the bulk diffusion timescale by $t_{d}$ and the surface dissolution timescale by $t_{s}$. For the fine grind parameters in [5] the values of the timescales are $t_{s}={1.184}~\mbox{s}$ and $t_{d}={42.231}~\mbox{s}$. For the coarse grind parameters the values of the timescales are $t_{s}={19.389}~\mbox{s}$ and $t_{d}={270.493}~\mbox{s}$. To begin we scale the equations on the slow timescale. We will presently see that the problem is of a boundary layer type, and using the diffusion timescale is equivalent to an outer scaling. The concentration scale for the fluid surrounding the grains is chosen by balancing the increase in concentration per unit time with the transport of coffee solubles from the kernels of the grain by diffusion. The concentration scale in the intragranular pores is chosen to be equal to the coffee solubility in water $c_{\mathrm{sat}}$. The scale for $\psi_{s}^{*}$ can be chosen either from the initial condition $\psi_{s}^{*}(0) = \psi_{s0}^{*}=1$ or by balancing the source term for the transfer of coffee from the grain surfaces with the other two terms in equation (18). Either of these scales allow us to solve the problem so we adopt the former which is more tractable. Thus the scales are

21 $$\begin{aligned}& c_{h}^{*} \sim\frac{c_{\mathrm{sat}}\phi_{v}^{\infty}(1-\phi _{h})}{\phi_{h}} , \qquad c_{v}^{*} \sim c_{\mathrm{sat}}, \end{aligned}$$

22 $$\begin{aligned}& t^{*} \sim t_{d} = \frac{k_{sv2} l_{l}}{6 \alpha^{*}{\phi_{v}^{\infty }}^{\frac{1}{3}}D_{v}}, \qquad \psi_{s}^{*} \sim\psi_{s0}^{*}=1. \end{aligned}$$

Before the dimensionless equations are presented we tidy up the presentation by introducing some dimensionless parameters. First the ratio of the surface dissolution timescale to the timescale of diffusion of coffee from the grain kernels is denoted by

23 $$ \epsilon= \frac{\alpha^{*} D_{v} k_{sv1} m {\phi_{v}^{\infty }}^{\frac{1}{3}} c_{s}}{2 \beta^{*} D_{h} k_{sv2} l_{l} \phi_{cd} c_{\mathrm{sat}} r_{s}}. $$

Also we introduce

24 $$ b_{1} = \frac{2 \beta^{*} D_{h} k_{sv2} l_{l} \phi_{cd} {\phi _{v}^{\infty}}^{\frac{2}{3}}(1-\phi_{h}) c_{\mathrm{sat}} r_{s}}{\alpha ^{*} D_{v} k_{sv1} m \phi_{h} c_{s}}, \qquad b_{2} = \frac{2 \beta^{*} D_{h} k_{sv2} l_{l} \phi_{cd} (1-\phi_{h})}{\alpha^{*} D_{v} k_{sv1} m \phi_{h} {\phi_{v}^{\infty}}^{\frac{1}{3}}}. $$

For parameters of both fine and coarse grinds the parameter $\epsilon \ll1$, while $b_{1}$ and $b_{2}$ are both $O(1)$. We represent the non-dimensional initial concentration of coffee in the intragranular pores by the parameter $\gamma_{1}$. Thus our equations and initial conditions on the diffusion timescale are

25 $$\begin{aligned}& \epsilon\frac{d C_{h}}{d \tau} =-\epsilon b_{2} C_{h} \Psi_{s}+\frac {b_{2}}{b_{1}}\Psi_{s}-\epsilon^{2} b_{1} C_{h} +\epsilon C_{v}, \end{aligned}$$

26 $$\begin{aligned}& \frac{d C_{v}}{d \tau} = \epsilon b_{1} C_{h}- C_{v}, \end{aligned}$$

27 $$\begin{aligned}& \epsilon\frac{d \Psi_{s}}{d \tau} = \epsilon b_{1} C_{h} \Psi_{s} -\Psi _{s}, \end{aligned}$$

28 $$\begin{aligned}& C_{h}(0) =0, \qquad C_{v}(0) = \gamma_{1}, \qquad \Psi_{s}(0) = 1. \end{aligned}$$

On the long timescale, this suggests that at leading order $\Psi_{s}$ relaxes to a steady state zero value, while $C_{v}$ decays exponentially with time. It is clear from the form of the equations that we have a singular perturbation and so initial conditions may not be applied to the outer solutions. For this system we have a number of ways to solve the problem. To begin with we consider the solution of the problem in a phase plane.

### Phase plane analysis

In the case of extraction of coffee from a suspension of coffee grains, unlike the system for extraction from a packed coffee bed, we have conservation of coffee in the system. Thus we can reduce the number of equations in the system by one by using this property. We note that

29 $$ \frac{d C_{h}}{d \tau} + \frac{d C_{v}}{d \tau} +\frac {b_{2}}{b_{1}}\frac{d \Psi_{s}}{d \tau}=0, $$

and integrating with respect to *τ* gives

30 $$ C_{h}(\tau) + C_{v}(\tau) +\frac{b_{2}}{b_{1}} \Psi_{s}(\tau)=C, $$

with *C* constant. Using the initial conditions we can write

31 $$ \Psi_{s}(\tau)=1 -\frac{b_{1}}{b_{2}} \bigl( C_{h}(\tau) + C_{v}(\tau )-\gamma_{1} \bigr). $$

**Figure 4:**
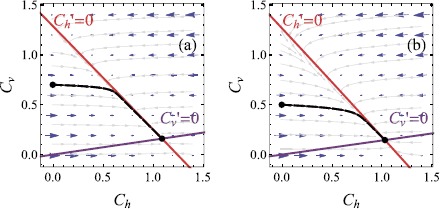
**Phase plane solutions.** The phase plane diagrams are given for **(a)** fine grind parameters $\epsilon= 0.028$, $b_{1} = 5.239$, $b_{2} =2.897$ and $\gamma_{1}=0.70$ (JK drip filter grind) and **(b)** coarse grind parameters $\epsilon= 0.071$, $b_{1} = 1.99$, $b_{2} =1.35$ and $\gamma_{1}=0.5$ (Cimbali #20 grind). Solution trajectories and direction fields are plotted in both cases. The magnitude of the direction field is represented by the size of the blue direction vectors.

Thus $\Psi_{s}$ can be eliminated from the system to give

32 $$\begin{aligned}& \epsilon\frac{d C_{h}}{d \tau} = \biggl(\frac{b_{2}}{b_{1}}-\epsilon b_{2} C_{h} \biggr) \biggl( 1 -\frac{b_{1}}{b_{2}} ( C_{h} + C_{v}-\gamma_{1} ) \biggr)-\epsilon^{2} b_{1} C_{h} +\epsilon C_{v}, \end{aligned}$$

33 $$\begin{aligned}& \frac{d C_{v}}{d \tau} = \epsilon b_{1} C_{h}- C_{v}, \end{aligned}$$

with initial conditions given by (28). Writing the equations in this form has some advantages. The number of equations is reduced to two, which facilitates the investigation of the solutions for $C_{h}$ and $C_{v}$ on a phase plane. We find the nullclines by setting the right-hand sides of equations (32) and (33) equal to zero. The physically relevant region of interest in the problem is $C_{h}>0$, $C_{v}>0$ and $C_{h}+C_{v}<\frac{b_{2}}{b_{1}} +\gamma_{1}$. Once the nullclines are drawn on the phase plane it is relatively straightforward to plot the solution on the plane. The phase planes and solution trajectories for the fine and coarse grind experiments are shown in Figure 4. In both cases, we see a rapid transition from the initial point to the $C_{h}$ nullcline before a much slower transition along the slow manifold towards a stable equilibrium point at the intersection of the nullclines. This stable equilibrium is given by

34 $$ C_{h} = \frac{b_{1} \gamma_{1}+b_{2}}{b_{1} (b_{1} \epsilon+1 )},\qquad C_{v} = \frac{\epsilon (b_{1} \gamma_{1}+b_{2} )}{b_{1} \epsilon+1}. $$

It is clearly useful to be able to reduce the number of equations to two, but it is more difficult to understand the physical meaning of the individual terms when the equation is written in this from. Thus when proceeding with the determination of approximate solutions of the problem the system of three differential equations will be used.

### Perturbation solutions on the bulk diffusion (outer) timescale

We use the following expansions for the bulk diffusion timescale

35 $$\begin{aligned}& C_{h} \sim C_{h0} + \epsilon C_{h1} + \epsilon^{2} C_{h2}, \end{aligned}$$

36 $$\begin{aligned}& C_{V} \sim C_{v0} + \epsilon C_{v1} + \epsilon^{2} C_{v2}, \end{aligned}$$

37 $$\begin{aligned}& \Psi_{s} \sim\Psi_{s0} + \epsilon\Psi_{s1} + \epsilon^{2} \Psi_{s2}. \end{aligned}$$

Substituting these expansions into the equations (25)-(27) collecting terms at each order, it is straightforward to find the solutions on the bulk diffusion timescale. As mentioned above the problem is singularly perturbed. Thus these outer solutions will involve constants which need to be determined by matching solutions obtained from the rescaling of the problem to the initial layer.

#### Leading order equations

The leading order equations are

38 $$\begin{aligned}& \Psi_{s0} =0, \end{aligned}$$

39 $$\begin{aligned}& \frac{d C_{v0}}{d \tau} = 0, \end{aligned}$$

40 $$\begin{aligned}& \Psi_{s0} =0. \end{aligned}$$

As expected we see that $\Psi_{s0}=0$. Solving the second equation we find that

41 $$ C_{v0}(\tau) =K_{1} e^{-\tau}. $$

#### Order *ϵ* equations

Substituting in the known terms, the order *ϵ* equations are

42 $$\begin{aligned}& \frac{d C_{h0}}{d \tau} = \frac{b_{2}}{b_{1}}\Psi_{s1}+K_{1} e^{-\tau}, \end{aligned}$$

43 $$\begin{aligned}& \frac{d C_{v1}}{d \tau} = b_{1}C_{h0}-C_{v1}, \end{aligned}$$

44 $$\begin{aligned}& \Psi_{s1} =0. \end{aligned}$$

Once again we see immediately that $\Psi_{s1}=0$. Solving the remaining two equations we find that

45 $$\begin{aligned}& C_{h0}(\tau) = K_{2}-K_{1} e^{-\tau}, \end{aligned}$$

46 $$\begin{aligned}& C_{v1}(\tau) = b_{1} e^{-\tau}\bigl(K_{2} e^{\tau}-K_{1} \tau\bigr)-K_{3} e^{-\tau}. \end{aligned}$$

#### Order $\epsilon^{2}$ equations

Substituting in the known terms, the order $\epsilon^{2}$ equations are

47 $$\begin{aligned}& \frac{d C_{h1}}{d \tau} = \frac{b_{2}}{b_{1}}\Psi_{s2}+K_{1}b_{1} e^{-\tau}-K_{1} b_{1} \tau e^{-\tau}+K_{3} e^{-\tau}, \end{aligned}$$

48 $$\begin{aligned}& \frac{d C_{v2}}{d \tau} = b_{1}\bigl(K_{2}-K_{1} e^{-\tau}\bigr)-C_{v2}, \end{aligned}$$

49 $$\begin{aligned}& \Psi_{s2} =0. \end{aligned}$$

Solving the non-trivial equations we find that

50 $$\begin{aligned}& C_{h1}(\tau) = e^{-\tau} (b_{1}K_{1} \tau-K_{3})+K_{4}, \end{aligned}$$

51 $$\begin{aligned}& C_{v2}(\tau) = b_{1} e^{-\tau} \biggl( \frac{1}{2} b_{1} K_{1} \tau^{2}-K_{3} \tau+K_{4} e^{\tau} \biggr)+K_{5} e^{-\tau}. \end{aligned}$$

#### Outer solutions

Collecting the outer solutions we have

52 $$\begin{aligned}& C_{h}(\tau) = K_{2}-K_{1} e^{-\tau}+ \epsilon \bigl(e^{-\tau} (b_{1}K_{1} \tau-K_{3})+K_{4} \bigr), \end{aligned}$$

53 $$\begin{aligned}& C_{v}(\tau) = K_{1} e^{-\tau}+\epsilon \bigl(b_{1} e^{-\tau}\bigl(K_{2} e^{\tau}-K_{1} \tau\bigr)-K_{3} e^{-\tau} \bigr) \\& \hphantom{C_{v}(\tau) ={}}{}+\epsilon^{2} \biggl(b_{1} e^{-\tau} \biggl(\frac{1}{2} b_{1} K_{1} \tau ^{2}-K_{3} \tau+K_{4} e^{\tau} \biggr)+K_{5} e^{-\tau} \biggr) , \end{aligned}$$

54 $$\begin{aligned}& \Psi_{s}(\tau) \equiv0, \end{aligned}$$

where $K_{1}$-$K_{5}$ are constants to be determined by matching with the inner. The outer solution for $\Psi_{s}(\tau)$ is identically zero.

### Perturbation solutions on the surface dissolution (inner) timescale

In this section we will consider the system behaviour in the initial layer. To do this we rescale time to the fast timescale, by introducing an initial layer coordinate $t=\frac{\tau}{\epsilon}$. We also rescale $c_{h}^{*}$ and to account for the different balances in the system in the initial layer. In particular the concentration scale for $c_{h}$ is chosen to balance the rate of change of concentration with the surface dissolution term. The scale for $\psi_{s}^{*}$ is again chosen as its initial value. Specifically we rescale the system as follows:

55 $$\begin{aligned}& \tau = \epsilon t, \qquad C_{h}(\tau) = \frac{b_{2}}{b_{1}}c_{h}(t), \end{aligned}$$

56 $$\begin{aligned}& C_{v}(\tau) = c_{v}(t), \qquad \Psi_{s}(\tau) = \psi_{s}(t). \end{aligned}$$

The scales for each of the dimensional variables in the initial layer are

57 $$\begin{aligned}& c_{h}^{*} \sim\frac{c_{s} (1-\phi_{h})}{r_{s} \phi_{h}}c_{\mathrm{sat}},\qquad c_{v}^{*} \sim c_{\mathrm{sat}}, \end{aligned}$$

58 $$\begin{aligned}& t^{*} \sim t_{s}=\frac{c_{s} k_{sv1} m}{12 \beta^{*} c_{\mathrm{sat}} D_{h} \phi_{cd} r_{s} }, \qquad \psi_{s}^{*} \sim{\psi_{s}^{*}}_{0}=1. \end{aligned}$$

Thus the equations on the inner timescale are given by:

59 $$\begin{aligned}& \frac{d c_{h}}{d t} =-\epsilon b_{2} c_{h} \psi_{s}+\psi_{s}-\epsilon^{2} b_{1} c_{h} +\epsilon\frac{b_{1}}{b_{2}} c_{v}, \end{aligned}$$

60 $$\begin{aligned}& \frac{d c_{v}}{d t} = \epsilon^{2} b_{2} c_{h}- \epsilon c_{v}, \end{aligned}$$

61 $$\begin{aligned}& \frac{d \psi_{s}}{d t} = \epsilon b_{2} c_{h} \psi_{s} -\psi_{s}, \end{aligned}$$

62 $$\begin{aligned}& c_{h}(0) = 0,\qquad c_{v}(0) = \gamma_{1}, \qquad \psi_{s}(0) =1. \end{aligned}$$

It is apparent that at leading order on the fast timescale $\psi_{s0}$ decays exponentially, while $c_{v}$ is constant. To find the solutions in detail we use the following regular expansions for the surface dissolution timescale:

63 $$\begin{aligned}& c_{h} \sim c_{h0} + \epsilon c_{h1} + \epsilon^{2} c_{h2}, \end{aligned}$$

64 $$\begin{aligned}& c_{v} \sim c_{v0} + \epsilon c_{v1} + \epsilon^{2} c_{v2}, \end{aligned}$$

65 $$\begin{aligned}& \psi_{s} \sim\psi _{s0} + \epsilon\psi_{s1} + \epsilon^{2} \psi_{s2}. \end{aligned}$$

#### Leading order equations

The leading order equations are

66 $$\begin{aligned}& \frac{d c_{h0}}{d t} = \psi_{s0}, \end{aligned}$$

67 $$\begin{aligned}& \frac{d c_{v0}}{d t} = 0, \end{aligned}$$

68 $$\begin{aligned}& \frac{d \psi_{s0}}{d t} = -\psi_{s0}, \end{aligned}$$

69 $$\begin{aligned}& c_{h0}(0) = 0, \qquad c_{v0}(0) = \gamma_{1}, \qquad \psi_{s0}(0) =1. \end{aligned}$$

Solving these equations we find that

70 $$\begin{aligned}& c_{h0}(t) = 1-e^{-t}, \end{aligned}$$

71 $$\begin{aligned}& c_{v0}(t) = \gamma_{1}, \end{aligned}$$

72 $$\begin{aligned}& \psi_{s0}(t) = e^{-t}. \end{aligned}$$

#### Order *ϵ* equations

The order *ϵ* equations are

73 $$\begin{aligned}& \frac{d c_{h1}}{dt} = \psi_{s1}-b_{2}c_{h0} \psi_{s0} + \frac {b_{1}}{b_{2}}c_{v0} , \end{aligned}$$

74 $$\begin{aligned}& \frac{d c_{v1}}{dt} =-c_{v0}, \end{aligned}$$

75 $$\begin{aligned}& \frac{d \psi_{s1}}{dt} = b_{2}c_{h0}\psi_{s0}- \psi_{s1}, \end{aligned}$$

76 $$\begin{aligned}& c_{h1}(0) = 0, \qquad c_{v1}(0) = 0, \qquad \psi_{s1}(0) =0. \end{aligned}$$

Substituting in the leading order solutions we can solve these equations to obtain

77 $$\begin{aligned}& c_{h1}(t) = \frac{b_{1} \gamma_{1} t}{b_{2}}+b_{2} e^{-2 t} \bigl(-e^{t} (t-1)-1 \bigr), \end{aligned}$$

78 $$\begin{aligned}& c_{v1}(t) = -\gamma_{1} t, \end{aligned}$$

79 $$\begin{aligned}& \psi_{s1}(t) = b_{2} e^{-2 t} \bigl(e^{t} (t-1)+1 \bigr). \end{aligned}$$

#### Order $\epsilon^{2}$ equations

The order $\epsilon^{2}$ equations are

80 $$\begin{aligned}& \frac{d c_{h2}}{dt} = \psi_{s2}-b_{2}(c_{h1} \psi_{s0}+c_{h0}\psi _{s1})+b_{1}c_{h0} + \frac{b_{1}}{b_{2}}c_{v1} , \end{aligned}$$

81 $$\begin{aligned}& \frac{d c_{v2}}{dt} =b_{2}c_{h0}-c_{v1}, \end{aligned}$$

82 $$\begin{aligned}& \frac{d \psi_{s2}}{dt} = b_{2}(c_{h1}\psi_{s0}+c_{h0} \psi _{s1})-\psi_{s2}, \end{aligned}$$

83 $$\begin{aligned}& c_{h2}(0) = 0, \qquad c_{v2}(0) = 0, \qquad \psi_{s2}(0) =0. \end{aligned}$$

Substituting in the known terms we can solve the equations to obtain

84 $$\begin{aligned}& c_{h2}(t) = \frac{e^{-3 t} (-b_{1} b_{2} e^{2 t} (\gamma_{1} t^{2}+2 e^{t} (t-1)+2 ) )}{ b_{2}} \\& \hphantom{c_{h2}(t) ={}}{}+ \frac{e^{-3 t} (-b_{1} \gamma_{1} e^{3 t} t^{2}-b_{2}^{3} (e^{t} (e^{t} (t-2) t+4 t-2 )+2 ) )}{2 b_{2}}, \end{aligned}$$

85 $$\begin{aligned}& c_{v2}(t) = e^{-3 t} \bigl(b_{1} \gamma_{1} e^{2 t} t^{2}+b_{2}^{2} \bigl(e^{t} \bigl(e^{t} (t-2) t+4 t-2 \bigr)+2 \bigr) \bigr), \end{aligned}$$

86 $$\begin{aligned}& \psi_{s2}(t) = \frac{1}{2} \bigl(e^{-3 t} \bigl(b_{1} \gamma_{1} e^{2 t} t^{2}+b_{2}^{2} \bigl(e^{t} \bigl(e^{t} (t-2) t+4 t-2 \bigr)+2 \bigr) \bigr) \bigr). \end{aligned}$$

#### Inner solutions

Collecting the solutions of each order we have the following inner solutions:

87 $$\begin{aligned}& c_{h}(t) = e^{-t} \bigl(e^{t}-1 \bigr)+\epsilon \biggl(\frac{b_{1} \gamma _{1} t}{b_{2}}+b_{2} e^{-2 t} \bigl(-e^{t} (t-1)-1 \bigr) \biggr) \\& \hphantom{c_{h}(t) ={}}{}+\epsilon^{2} \biggl(\frac{e^{-3 t} (-b_{1} b_{2} e^{2 t} (\gamma_{1} t^{2}+2 e^{t} (t-1)+2 ) )}{ b_{2}} \\& \hphantom{c_{h}(t) ={}}{}+\frac{e^{-3 t} (-b_{1} \gamma_{1} e^{3 t} t^{2}-b_{2}^{3} (e^{t} (e^{t} (t-2) t+4 t-2 )+2 ) )}{2 b_{2}}\biggr), \end{aligned}$$

88 $$\begin{aligned}& c_{v}(t) = \gamma_{1}-\epsilon(\gamma_{1} t)+ \epsilon^{2} \biggl(b_{2} \bigl(t+e^{-t}-1 \bigr)+ \frac{\gamma_{1} t^{2}}{2} \biggr), \end{aligned}$$

89 $$\begin{aligned}& \psi_{s}(t) =e^{-t}+\epsilon \bigl(b_{2} e^{-2 t} \bigl(e^{t} (t-1)+1 \bigr) \bigr) \\& \hphantom{\psi_{s}(t) ={}}{}+\frac{1}{2} \epsilon^{2} \bigl(e^{-3 t} \bigl(b_{1} \gamma_{1} e^{2 t} t^{2}+b_{2}^{2} \bigl(e^{t} \bigl(e^{t} (t-2) t+4 t-2 \bigr)+2 \bigr) \bigr) \bigr). \end{aligned}$$

### Matching

The integration constants in the outer solutions can be determined by matching these solutions with the initial layer solutions. We do this using modified Van Dyke matching [24]. In this method we compare the inner expansion of the outer solutions with the outer expansion of the inner solutions to obtain the unknown constants $K_{1}$-$K_{5}$. Applying this method we find that

90 $$\begin{aligned}& K_{1} = \gamma_{1}, \qquad K_{2} = \frac{b_{2}}{b_{1}}+\gamma_{1}, \qquad K_{3} =-b_{1} \gamma_{1}-b_{2}, \end{aligned}$$

91 $$\begin{aligned}& K_{4} = -b_{1} \gamma_{1}-b_{2}, \qquad K_{5} = b_{1}^{2} \gamma_{1}+b_{2} b_{1}-b_{2}. \end{aligned}$$

The matched outer solutions are

92 $$\begin{aligned}& C_{h}(\tau) = \frac{b_{2}}{b_{1}}-\gamma_{1} e^{-\tau}+\gamma_{1}+ \epsilon \bigl(e^{-\tau} \bigl(b_{1} \gamma_{1} (\tau+1)+b_{2} \bigr)-(b_{1} \gamma _{1}+b_{2}) \bigr), \end{aligned}$$

93 $$\begin{aligned}& C_{v}(\tau) = \gamma_{1} e^{-\tau}+ \epsilon \bigl(e^{-\tau} \bigl(b_{1} \gamma_{1} e^{\tau}-b_{1} \gamma_{1} \tau+b_{2} e^{\tau} \bigr)-e^{-\tau} (b_{1} \gamma_{1}+b_{2}) \bigr) \\& \hphantom{C_{v}(\tau) ={}}{}+\epsilon^{2} \biggl(e^{-\tau} \bigl(b_{1}^{2} \gamma_{1}+b_{1} b_{2}-b_{2} \bigr) \\& \hphantom{C_{v}(\tau) ={}}{}-b_{1} e^{-\tau} \biggl(-\frac{1}{2} b_{1} \gamma_{1} \tau^{2}-b_{1} \gamma_{1} \tau+b_{1} \gamma_{1} e^{\tau}-b_{2} \tau+b_{2} e^{\tau} \biggr) \biggr), \end{aligned}$$

94 $$\begin{aligned}& \Psi_{s}(\tau) = 0. \end{aligned}$$

Thus the composite solutions on the inner timescale and concentration scale are

95 $$\begin{aligned}& {c_{h}}_{\mathrm{comp}}(t) = c_{h}(t)+ \frac{b_{1}}{b_{2}}C_{h}(\epsilon t)-\frac {\epsilon^{2} (-b_{1} b_{2} t-\frac{1}{2} b_{1} \gamma_{1} t^{2} )+b_{1} \gamma1 t \epsilon+b_{2}}{b_{2}}, \end{aligned}$$

96 $$\begin{aligned}& {c_{v}}_{\mathrm{comp}}(t) = c_{v}(t)+C_{v}( \epsilon t)- \biggl(\epsilon^{2} \biggl(b_{2} t-b_{2}+\frac{\gamma_{1} t^{2}}{2} \biggr)+\gamma_{1}- \gamma_{1} t \epsilon \biggr), \end{aligned}$$

97 $$\begin{aligned}& {\psi_{s}}_{\mathrm{comp}}(t) = \psi_{s}(t). \end{aligned}$$

### Comparison of asymptotics with numerical simulation

**Figure 5:**
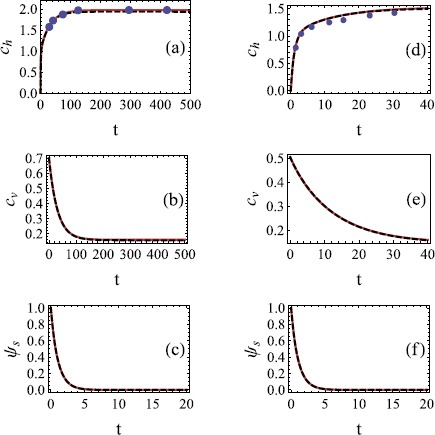
**Comparison of numerical and approximate solutions for fine and coarse grinds.** Comparison of numerical (-  -) and composite (—) solutions for **(a)** $c_{h}(t)$ (experimental data points are included), **(b)** $c_{v}(t)$ and **(c)** $\psi_{s}(t)$ for $\epsilon= 0.028$, $b_{1} = 5.239$, $b_{2} =2.897$ and $\gamma_{1}=0.70$ (JK drip filter grind). Plots **(d)**, **(e)** and **(f)** show the corresponding results for $\epsilon= 0.071$, $b_{1} = 1.99$, $b_{2} =1.35$ and $\gamma_{1}=0.5$ (Cimbali #20 grind).

The composite solutions are compared with the numerical solution of equations (59)-(62) and available experimental data in Figure 5. The comparison with data is shown for two different grind size distributions from [5]: one fine grind called JK drip filter grind and one relatively coarse grind called Cimbali #20 grind. Available data for coffee concentration in the intergranular fluid is compared to the approximate and numerical solutions based on the parameters for these two grinds. The asymptotic and numerical solutions to the model for intragranular concentration $c_{v}(t)$ and the fraction of coffee remaining on the grain surfaces $\psi_{s}(t)$ are also plotted. No data is available for comparison in this case.

## Conclusion

In this paper the mathematical model of coffee extraction described in [5] is analysed. The model equations are simplified and non-dimensionalised to describe extraction of coffee from a dilute suspension of coffee grains. The dimensionless form of the equations represents a much simpler description of the important processes in this situation and depends on a much smaller set of parameters than the original equations. This is a good example of model reduction [25]. Approximate solutions of the specialised dimensionless equations can be found using perturbation techniques. These solutions depend on dimensionless parameters which are defined in terms of the physical parameters of the system. Thus the approximate solutions are useful to both quickly fit solutions to a particular set of process parameters and to investigate the influence of changing a particular parameter on the system.

The model equations used to describe the extraction of coffee in a fixed water volume admit approximate solutions due to the existence of two different extraction mechanisms over different timescales. Extraction of coffee from the surfaces of the coffee grains proceeds much faster than diffusion of coffee through the intragranular pore network to the grain surface. The ratio of these timescales gives us a small dimensionless parameter. We utilise this parameter to form solutions on the inner (surface dissolution) and outer (diffusion from grain kernel) timescales based on the dominant mechanisms in these regimes. These approximate solutions are found to match the numerical solutions well and provide a much simpler equation to fit to data.

Extraction of coffee solubles from individual coffee grains is a key operation in many brewing techniques. The physical description of extraction here is observed to describe extraction well in the case of extraction from a dilute suspension of coffee grains. Thus it may be useful in models of more complicated brewing techniques to describe the grain extraction kinetics component of the procedure. This has already been shown in the case of extraction in flow through a packed coffee bed [5]. There is a wide variety of topics in the area of coffee extraction and coffee brewing in general which may benefit from further mathematical modelling and investigation. The model in [5] may be generalised to describe the unsaturated flow during the filling and draining of a coffee filter. The model can also easily be extended to model the extraction of a number of different coffee constituents rather than just a single entity. This may be useful if the influence on flavour of particular constituents (or groups of constituents which extract at similar rates) can be identified. The modelling of coffee in a drip filter presents a number of challenges. The filter geometry needs to be accounted for, the fluid flow in the filter may be complex and the coffee grains can be transported around the filter in the flow. On the scale of a coffee grain there is scope to investigate the dissolution of coffee within a coffee grain in more detail to investigate some of the assumptions made in this paper.

## References

[1] Farah A (2012). Coffee constituents. Coffee: emerging health effects and disease prevention.

[2] Petracco M (2008). Technology IV: beverage preparation: brewing trends for the new millennium. Coffee: recent developments.

[3] Pictet G, Clarke RJ, Macrae R (1987). Home and catering brewing of coffee. Coffee.

[4] Rao S. Everything but expresso: professional coffee brewing techniques. Scott Rao; 2010. Available from: www.scottrao.com.

[5] Moroney KM, Lee WT, O’Brien SBG, Suijver F, Marra J (2015). Modelling of coffee extraction during brewing using multiscale methods: an experimentally validated model. Chem Eng Sci.

[6] Sivetz M, Foote HE (1963). Coffee processing technology.

[7] Spaninks JAM. Design procedures for solid-liquid extractors and the effect of hydrodynamic instabilities on extractor performance [PhD thesis]. Agricultural University of Washington; 1979.

[8] Clarke RJ, Clarke RJ, Macrae R (1987). Extraction. Coffee.

[9] Navarini L, Nobile E, Pinto F, Scheri A, Suggi-Liverani F (2009). Experimental investigation of steam pressure coffee extraction in a stove-top coffee maker. Appl Therm Eng.

[10] Gianino C (2007). Experimental analysis of the Italian coffee pot ‘moka’. Am J Phys.

[11] Fasano A, Talamucci F (2000). A comprehensive mathematical model for a multispecies flow through ground coffee. SIAM J Math Anal.

[12] Fasano A, Farina A (2010). Modelling complex flows in porous media by means of upscaling procedures. Rend Ist Mat Univ Trieste.

[13] Fasano A, Talamucci F, Petracco M, Fasano A (2000). The espresso coffee problem. Complex flows in industrial processes.

[14] Fasano A, Fasano A (2000). Filtration problems in various industrial processes. Filtration in porous media and industrial application.

[15] Fasano A, Mikelić A (2000). On the filtration through porous media with partially soluble permeable grains. NoDEA Nonlinear Differ Equ Appl.

[16] Fasano A, Mikelić A, Primicerio M (1998). Homogenization of flows through porous media with permeable grains. Adv Math Sci Appl.

[17] Voilley A, Simatos D (1979). Modeling the solubilization process during coffee brewing. J Food Process Eng.

[18] Booth C, Cummins C, Dalwadi M, Dellar P, Devereux M, Dewynne J, Donohue J, Duncan A, Fitzmaurice F, Gordon A, Hennessy M, Hinch J, Hickey C, Hjorth P, Kyrke-Smith T, Leahy D, Lee W, Lynch E, Mercier O, Miklavcic S, Russell S, Schwartz L, Shozi BF, Swierczynski P, Timoney C, Tomczyk J, Warneford E. Brewing of filter coffee. Technical report from MACSI’s 2012 problem-solving workshop with industry; 2012. Available from: http://www.macsi.ul.ie/esgi87/ReportWeb.pdf.

[19] Schwartz LW (2014). An analysis of gravity-driven flow in a conical filter. J Eng Math.

[20] Moroney KM, Lee WT, O’Brien SBG, Suijver F, Marra J. Asymptotic analysis of the dominant mechanisms in the coffee extraction process. In preparation.

[21] Holdich RG (2002). Fundamentals of particle technology.

[22] Bear J, Cheng AH-D (2010). Modeling groundwater flow and contaminant transport.

[23] Jaganyi D, Madlala SP (2000). Kinetics of coffee infusion: a comparative study on the extraction kinetics of mineral ions and caffeine from several types of medium roasted coffees. J Sci Food Agric.

[24] Van Dyke M (1975). Perturbation methods in fluid mechanics.

[25] Schilders WH, van der Vorst HA, Rommes J (2008). Model order reduction: theory, research aspects and applications.

